# Accurate Prediction of Metachronous Liver Metastasis in Stage I-III Colorectal Cancer Patients Using Deep Learning With Digital Pathological Images

**DOI:** 10.3389/fonc.2022.844067

**Published:** 2022-04-01

**Authors:** Chanchan Xiao, Meihua Zhou, Xihua Yang, Haoyun Wang, Zhen Tang, Zheng Zhou, Zeyu Tian, Qi Liu, Xiaojie Li, Wei Jiang, Jihui Luo

**Affiliations:** ^1^ Department of General Surgery, Hunan Provincial People’s Hospital (The First-Affiliated Hospital of Hunan Normal University), Changsha, China; ^2^ Department of Microbiology and Immunology, Institute of Geriatric Immunology, School of Medicine, Jinan University, Guangzhou, China; ^3^ Department of Surgical Oncology, Chenzhou No. 1 People’s Hospital, Chenzhou, China; ^4^ Department of Pathology, Chenzhou No. 1 People’s Hospital, Chenzhou, China

**Keywords:** deep learning, colorectal cancer, metachronous liver metastasis, prediction model, nomogram

## Abstract

**Objectives:**

Metachronous liver metastasis (LM) significantly impacts the prognosis of stage I-III colorectal cancer (CRC) patients. An effective biomarker to predict LM after surgery is urgently needed. We aimed to develop deep learning-based models to assist in predicting LM in stage I-III CRC patients using digital pathological images.

**Methods:**

Six-hundred eleven patients were retrospectively included in the study and randomly divided into training (428 patients) and validation (183 patients) cohorts according to the 7:3 ratio. Digital HE images from training cohort patients were used to construct the LM risk score based on a 50-layer residual convolutional neural network (ResNet-50). An LM prediction model was established by multivariable Cox analysis and confirmed in the validation cohort. The performance of the integrated nomogram was assessed with respect to its calibration, discrimination, and clinical application value.

**Results:**

Patients were divided into low- and high-LM risk score groups according to the cutoff value and significant differences were observed in the LM of the different risk score groups in the training and validation cohorts (*P*<0.001). Multivariable analysis revealed that the LM risk score, VELIPI, pT stage and pN stage were independent predictors of LM. Then, the prediction model was developed and presented as a nomogram to predict the 1-, 2-, and 3-year probability of LM. The integrated nomogram achieved satisfactory discrimination, with C-indexes of 0.807 (95% CI: 0.787, 0.827) and 0.812 (95% CI: 0.773, 0.850) and AUCs of 0.840 (95% CI: 0.795, 0.885) and 0.848 (95% CI: 0.766, 0.931) in the training and validation cohorts, respectively. Favorable calibration of the nomogram was confirmed in the training and validation cohorts. Integrated discrimination improvement and net reclassification index indicated that the integrated nomogram was superior to the traditional clinicopathological model. Decision curve analysis confirmed that the nomogram has clinical application value.

**Conclusions:**

The LM risk score based on ResNet-50 and digital HE images was significantly associated with LM. The integrated nomogram could identify stage I-III CRC patients at high risk of LM after primary colectomy, so it may serve as a potential tool to choose the appropriate treatment to improve the prognosis of stage I-III CRC patients.

## Introduction

Colorectal cancer (CRC) is the third most common malignant cause of morbidity and mortality (1). Although the development of treatment strategies and multidisciplinary treatment has effectively reduced the recurrence rate, distant metastasis is still the main cause of the poor prognosis of patients with CRC (2, 3). Liver metastasis (LM) is the most common site for distant metastases because it is anatomically related to the portal circulation (4). Approximately 20%-40% of patients with CRC will develop metachronous LM after the initial surgery (5–7). Compared with other treatment methods, radical surgery is the main treatment scheme for LM detected early, which shows a better prognosis, providing these patients with a chance of cure (8, 9). However, a considerable number of patients with LM miss the opportunity for surgery when LM is discovered. Hence, it is important to screen patients at high-risk of developing LM and to detect LM early to improve the prognosis of stage I–III CRC patients. Currently, the management of CRC patients is mainly dependent on the tumor-node-metastasis (TNM) staging system, that is, the depth of tumor wall invasion (T), lymph node involvement (N), and distant metastasis (M). However, the traditional TNM staging system cannot effectively predict LM (10). Therefore, there is an urgent need for an effective biomarker to predict LM after surgery.

Recently, digital pathological images have attracted increased attention; they are scanned and collected by a fully automatic microscope or optical magnification system to obtain high-resolution digital images, and then a computer is used to automatically perform high-precision multifield seamless stitching and processing on the obtained images (11, 12). Moreover, digital pathological images provide a platform for deep learning that generally acknowledges that digital hematoxylin and eosin (HE) images contain valuable diagnostic and prognostic information (13–15). Since 2015, deep learning has become a powerful method that can automatically acquire the representation of essential disease features directly from images, thereby eliminating the process of manual feature engineering in traditional methods (16–19). Deep learning models have achieved human expert-level performance in multiple diagnostic applications involving medical image interpretation (16, 18). Importantly, deep learning has also shown good performance in predicting tumor prognosis (20, 21).

In this study, we aimed to construct an LM risk score based on digital HE images and deep learning to predict postoperative LM in stage I–III CRC patients who undergo radical resection. In addition, we developed and validated a nomogram that combined the LM risk score and clinicopathological predictors for the individual postoperative prediction of LM in stage I–III CRC patients.

## Materials and Methods

### Patients and Data Acquisition

**Figure 1:**
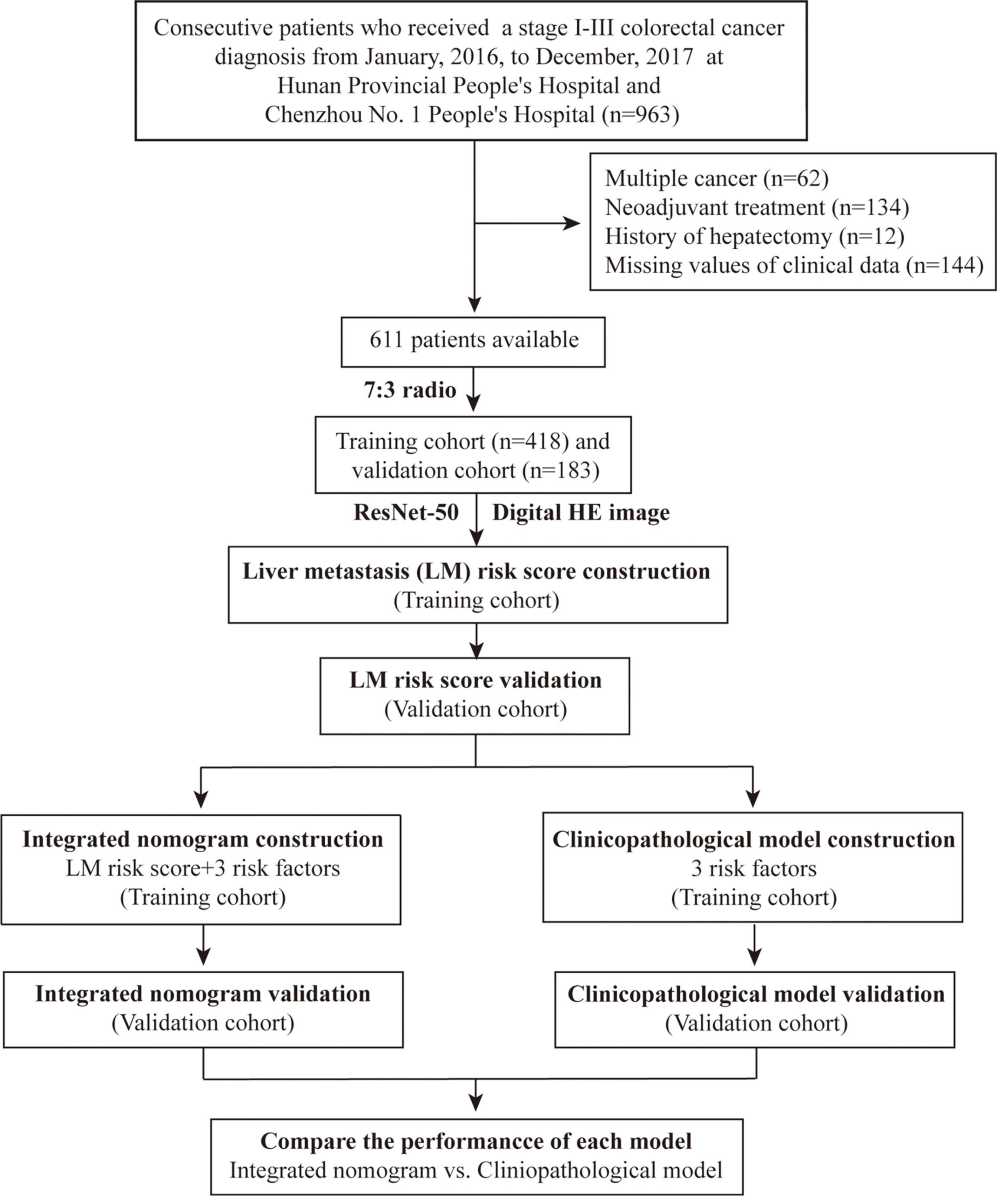
The overall process of this study.

We conducted a retrospective study on patients who underwent radical colorectal resection in Hunan Provincial People’s Hospital and Chenzhou No. 1 People’s Hospital from January 2016 to December 2017. Patients with stage I-III CRC who underwent radical resection were included in the study. The exclusion criteria included multiple primary cancers; preoperative neoadjuvant treatment; history of hepatectomy; and missing clinical data. Finally, 611 patients were included in the study. The patients were randomly divided into a training cohort (428 patients) and a validation cohort (183 patients) at a 7:3 ratio (**Figure 1**). This study was approved by the Institutional Review Boards of Hunan Provincial People’s Hospital and Chenzhou No. 1 People’s Hospital. Written informed consent was obtained from all patients. All procedures involving human participants were in accordance with the Declaration of Helsinki.

Patient baseline information, including age, sex, primary tumor location, preoperative carcinoembryonic antigen (CEA) level, preoperative cancer antigen 19-9 (CA 19-9) level, vascular emboli or lymphatic invasion or perineurial invasion (VELIPI), tumor differentiation, KRAS, BRAF, BRAS, PIK3CA, pT stage, pN stage, pTNM stage, and follow-up data (follow-up duration and survival status), was collected. TNM stage was reclassified according to the eighth edition of the American Joint Committee on Cancer (AJCC) Cancer Staging Manual.

All patients underwent the following follow-up examinations in the first 3 years after surgery: digital rectal and CEA examination every 3 months, liver ultrasound examination every six months, and colonoscopy and full abdominal computed tomography (CT) every year. The follow-up duration was measured from the time of surgery to the last follow-up date, and the survival status at the last follow-up was recorded.

### Digital Pathological Image Acquisition and Region of Interest Selection

All patient specimens were prepared with formalin-fixed paraffin-embedded tissue. The size of the specimen varied among subjects and thus, the size of the scanned images also varied. These specimens were stained with HE and scanned using an Aperio ImageScope (Lycra Biosystems, California, USA) at 20x magnification. After obtaining the patient’s digital HE image, a pathologist with 10 years of experience in the pathological diagnosis of CRC confirmed the tumor region as the region of interest (ROI) for the deep learning model, which was trained using the supervised learning method.

### Image Preprocessing

The ROI of each patient was split into patches of 1024 × 1024 μm in the training and validation cohorts. However, since the ROIs ranged from 1 to 2 GB, after screening patches with obvious interference factors (including bleeding, creases, necrosis, and blurred areas), the number of patches extracted from each patient was between 5,000 and 20,000. To save calculation time, we randomly selected 100 patches from each patient. Finally, after random cutting, random horizontal flipping, random affine transformation, center cropping, and normalization preprocessing, the patch was input into the deep learning model based on a residual convolutional neural network (ResNet).

### Transfer Learning of the 50-Layer Residual Neural Network

ResNet, as a branch of convolutional neural networks (CNNs), is currently one of the popular deep learning methods in the field of artificial intelligence (22). It uses feature transmission to prevent the gradient from disappearing to build a deeper neural network. Transfer learning is an effective method for applying these pretrained models to medical image analysis; thus, for LM prediction, we use the original network architecture of the ResNet-50 model, which divides 14 million labeled images from the ImageNet database into 1,000 object categories. First, all the patches were resized to 224 x 224 pixels for ResNet-50. Then, we fine-tuned the network, and all convolutional layers were fixed, which can significantly speed up network training and prevent overfitting to new medical data sets. The ResNet-50 network training was optimized using an Adam optimizer with 100 epochs and a learning rate of 0.0001 to ensure that the entire data set was covered for efficient training. The loss function was determined to be binary cross-entropy. We used the sigmoid function to calculate the probability before the output layer. Each patch would eventually produce a probability value between 0 and 1, and the average value of the 100 input patches as the LM risk score. The patches of the training cohort were trained through the pretrained ResNet-50 and verified with the patches of the validation cohort.

The ResNet-50 model was implemented with open-source Python (version 3.9.0) and TensorFlow (version 2.6.0-GPU) and was trained on a workstation equipped with a Core(TM) i5-10400F CPU @ 2.90 GHz (Intel; Santa Clara, CA) and one Nvidia GTX 1080 Ti GPU (Nvidia; Santa Clara, CA). The code that supports the findings of this study is available from the corresponding author upon reasonable request.

### Association of LM Risk Score With LM and Prognosis

The patients were classified into high- and low-LM risk score subgroups according to the optimal cutoff value, which was defined by the “survminer” R package (23) in the training cohort, and the same cutoff value was applied to the validation cohort. Kaplan–Meier survival analyses were conducted to assess the impacts of the LM risk score on LM, disease-free survival (DFS), and overall survival (OS). The “survminer” and “survival” packages were used to perform the survival analyses. DFS was defined as the time from surgery to recurrence at any site or all-cause death, whichever came first. OS was defined as the interval between surgery and death from any cause.

### Development and Validation of the Integrated Nomogram

The primary endpoint of the analysis was the time to postoperative LM. Univariate Cox regression analysis was conducted to assess the potential association of clinicopathological characteristics and the LM risk score with LM in the training cohort, and the hazard ratio (HR) with the corresponding 95% confidence interval (CI) was calculated. Variables with P < 0.05 in the univariate analyses were selected for the multivariate analysis. Finally, an integrated nomogram was developed based on the multivariate analysis results. A clinicopathological model containing only clinicopathological predictors was also constructed for comparison. Nomogram development was performed by the “rms” and “survival” packages.

The discrimination of the nomogram was measured by Harrell’s concordance index (C-index) (24, 25) and the time-dependent receiver operating characteristic (ROC) curve (26). The calibration curve was plotted to assess the agreement between the predicted and actual probabilities of LM. Decision curve analysis (DCA) was used to quantitatively analyze the clinical application value of the integrated nomogram (27). In addition, prediction errors over time (28, 29), net reclassification improvement (NRI), and integrated discrimination improvement (IDI) (30, 31) were calculated to compare the performance of the nomogram and the clinicopathological model. The ROC curves were plotted using the “timeROC” and “survival” packages. DCA was performed with the “dca.R” function. The prediction errors over time were assessed using the prediction error curves function of the “pec” package with the “Boot- 632plus” split method with 1000 iterations. The “survIDINRI” package was used for the calculation of NRI and IDI.

### Statistical Analysis

R software version 3.6.0 (https://www.r-project.org/) and SPSS software (version 22.0) were used for statistical analysis. Continuity variables were analyzed by t test, while categorical variables were analyzed by the χ2 test or Fisher’s exact test. Survival curves were generated by using Kaplan–Meier survival analysis, and the differences in survival distributions were tested using the log-rank test. Cox proportional risk regression models were used for univariate analysis and multivariate analysis. All tests were two-tailed, and a P value < 0.050 was determined to be statistically significant.

## Results

### Patient Demographics

Our study sample comprised 611 patients (392 males and 219 females) who underwent colectomy for stage I-III CRC. The mean patient age was 56.51 ± 12.00 years. The clinicopathological characteristics of the training cohort (n = 428) and validation cohort (n = 183) are listed in **Table 1**. The clinicopathological characteristics between the two cohorts were similar, which justifies the use of these cohorts as a training cohort and a validation cohort.

**Table 1:** Characteristics of the patients in the training and validation cohorts.

Variable	Training cohort N=428	Validation cohort N=183	*P*
**Age, years**	56.94 ± 11.77	56.07 ± 12.53	
**Sex**			0.508
Male	271 (63.3)	121 (66.1)	
Female	157 (36.7)	62 (33.9)	
**Primary tumor location**			0.638
Left-sided	328 (76.6)	137 (74.9)	
Right-sided	100 (23.4)	46 (25.1)	
**Preoperative CEA level**			0.060
Normal	241 (56.3)	118 (64.5)	
Elevated	187 (43.7)	65 (35.5)	
**Preoperative CA19-9 level**			0.219
Normal	344 (80.4)	139 (76.0)	
Elevated	84 (19.6)	44 (24.0)	
**VELIPI**			0.237
No	228 (53.3)	107 (58.5)	
Yes	200 (46.7)	76 (41.5)	
**Tumor differentiation**			0.830
Well or moderately	347 (81.1)	147 (80.3)	
Poorly or undifferentiated	81 (18.9)	36 (19.7)	
**KRAS**			0.491
Wild type	292 (68.2)	130 (71.0)	
Mutation	136 (31.8)	53 (29.0)	
**BRAF**			0.200
Wild type	389 (90.9)	172 (94.0)	
Mutation	39 (9.1)	11 (6.0)	
**BRAS**			0.734
Wild type	403 (94.2)	171 (93.4)	
Mutation	25 (5.8)	12 (6.6)	
**PIK3CA**			0.791
Wild type	373 (87.2)	157 (85.8)	
Mutation	66 (12.9)	26 (14.2)	
**pT stage**			0.738
I-II	167 (39.0)	70 (38.3)	
III	184 (43.0)	84 (45.9)	
IV	77 (18.0)	29 (15.8)	
**pN stage**			0.456
0	265 (61.9)	107 (58.5)	
I	99 (2301)	51 (27.9)	
II	64 (15.0)	25 (13.7)	
**TNM stage**			0.321
I	121 (28.3)	41 (22.4)	
II	144 (33.6)	66 (36.1)	
III	163 (38.1)	76 (41.5)	
**LM risk score**	0.404 ± 0.101	0.415 ± 0.100	0.224

CEA, carcinoembryonic antigen; CA19-9, carbohydrate antigen 19-9; VELIPI, vascular emboli or lymphatic invasion or perineurial invasion.

**Figure 2:**
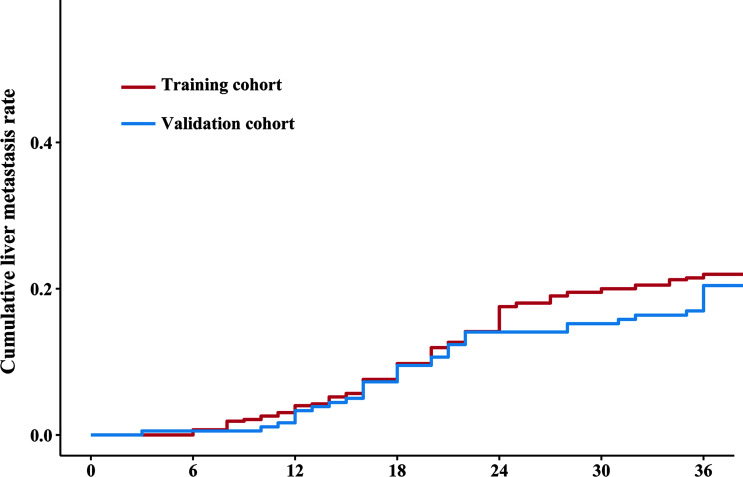
Cumulative liver metastasis rate in the training and validation cohorts. The red line and blue line indicate the cumulative liver metastasis rate in the training and validation cohorts, respectively. The cumulative rates of liver metastasis were 21.5% (92/418) and 19.7% (36/183) in the training and validation cohorts, respectively.

The median follow-up duration (IQR) was 39 (32–38) and 40 (32–38) months in the training and validation cohorts, respectively. The 3-year DFS and OS rates were 73.9% and 82.5% (**Supplementary Figures S1A, B**), respectively, in the training cohort, and 92 (21.5%) patients had LM after initial surgery (**Figure 2**). In the validation cohort, the 3-year DFS and OS rates were 73.8% and 83.1% (**Supplementary Figures S1C, D**), respectively, and 36 (19.7%) patients had LM (**Figure 2**).

### Training and Validation of the Deep Learning Model

**Figure 3:**
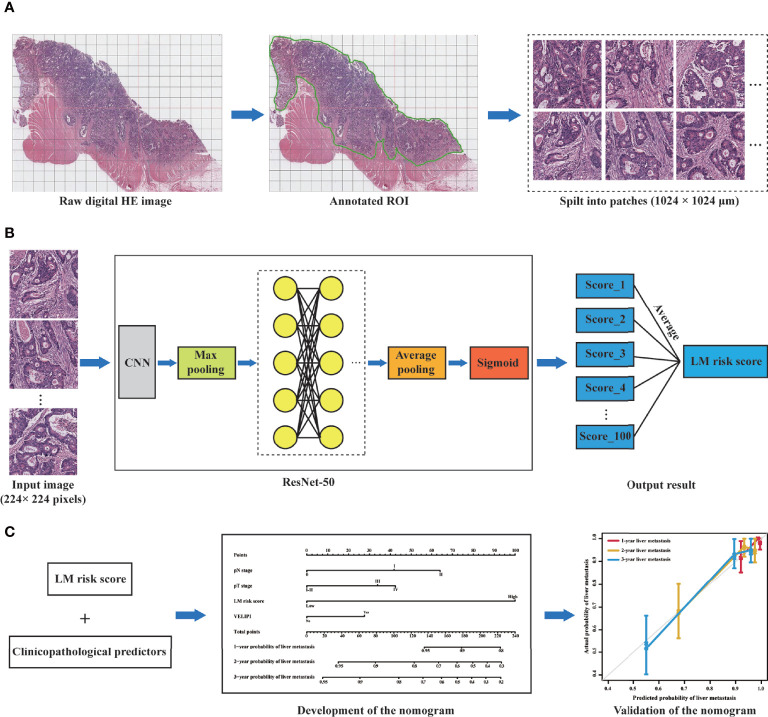
Workflow of this study. **(A)** Selection of the ROI on the digital HE image. The tumor ROI was then segmented into patches of 1024 × 1024 μm. **(B)** A total of 100 patches were randomly selected from each patient, and the liver metastasis likelihood of each patch was predicted by a deep learning model based on ResNet-50. Then, the probability values of the 100 patches were merged to generate an average value as the LM risk score. **(C)** A nomogram was developed based on the LM risk score and clinicopathological predictors in the training cohort and verified in the validation cohort. ROI, region of interest; CNN, convolutional neural network; ResNet-50, 50-layer residual network; LM, liver metastasis.

The workflow of this study is displayed in **Figure 3**. All the patches were augmented and trained in the training cohort *via* the ResNet-50 model to increase the robustness (**Supplementary Figure S2**). There was no significant difference in the LM risk score (mean ± SD) between the training (0.404 ± 0.101) and validation cohorts (0.415 ± 0.100) [*P* = 0.224] (**Table 1**). The ResNet-50 activation maps for high and low LM risk scores, which reflect the weights corresponding to the LM risk, were obtained from the digital HE images (**Supplementary Figure S3**).

**Figure 4:**
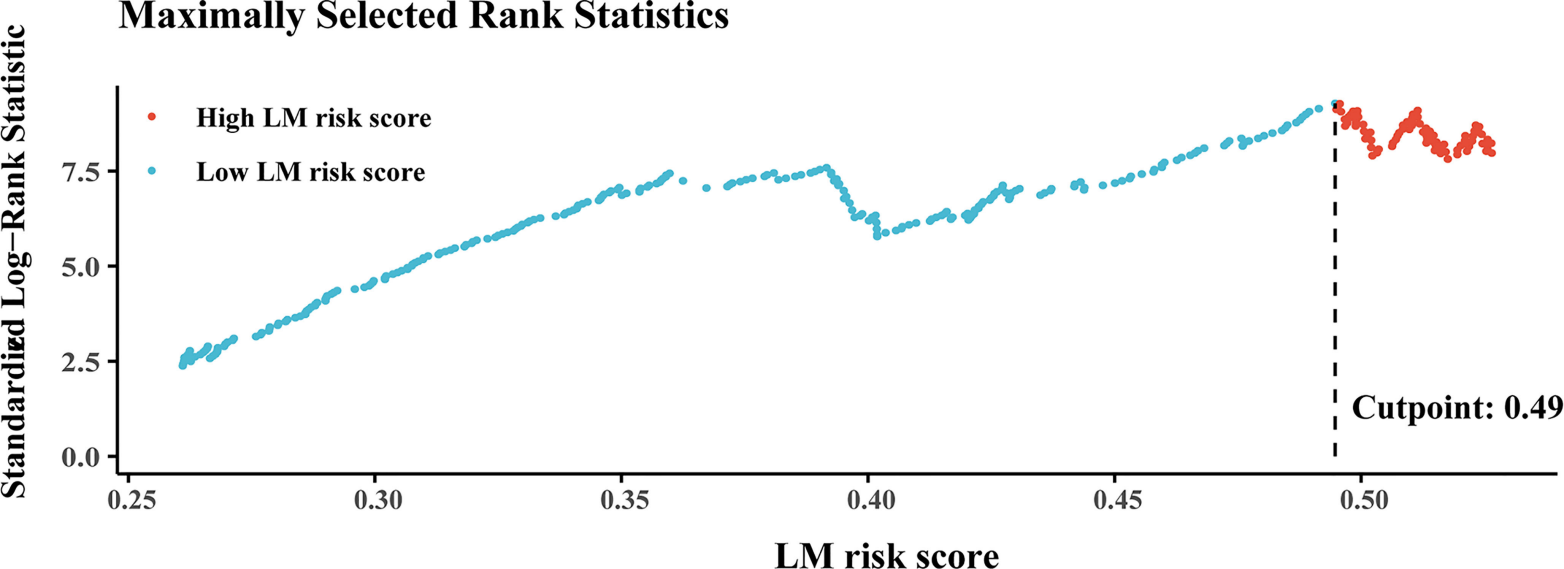
Plots of the best cutoff value of the LM risk score in the training cohort using the Kaplan–Meier method. LM, liver metastasis.

The best cutoff value generated by the “survival” R package was 0.49 (**Figure 4**) in the training cohort, and all patients were divided into high- and low-LM risk score subgroups. The LM risk scores of patients in the training and validation cohorts were calculated and are shown in **Supplementary Figure S4**. The clinicopathological characteristics according to the high- and low-LM risk score groups in the training and validation cohorts are presented in **Supplementary Table S1**.

**Figure 5:**
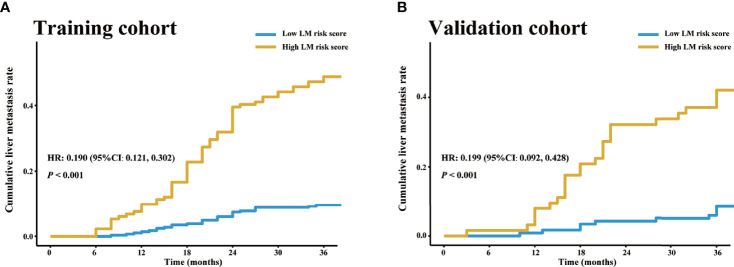
LM risk score and LM in the training and validation cohorts. Cumulative LM rate stratified by the LM risk score in the **(A)** training and **(B)** validation cohorts. The cumulative liver metastasis rates in the patients with high LM risk score were 48.1% (64/133) and 41.3% (26/63) in the training and validation cohorts, respectively, and the cumulative liver metastasis rates in the patients with low LM risk score were 9.5% (28/295) and 8.3% (10/120) in the training and validation cohorts, respectively. HR, hazard ratio; CI, confidence interval; LM, liver metastasis.

**Figure 6:**
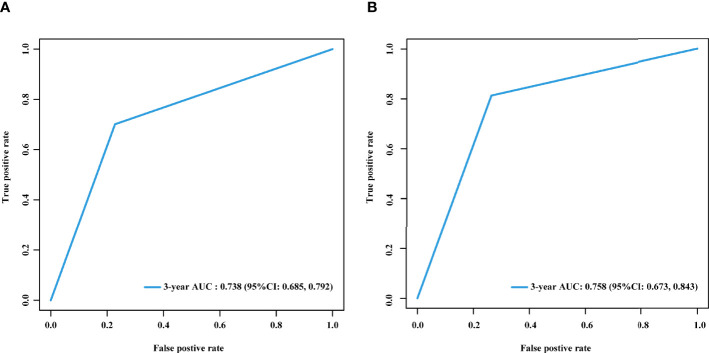
Time-dependent ROC curves of the LM risk score in the training and validation cohorts. Time-dependent ROC curves of the LM risk score in the training **(A)** and validation **(B)** cohorts at 3 years. AUC, area under the ROC curve; ROC, receiver operating characteristic; LM, liver metastasis; CI, confidence interval.

There was a significantly higher 3-year cumulative LM rate in patients with a high LM risk score than in those with a low LM risk score in the training cohort (48.1% *vs*. 9.5%; log-rank *P*<0.001) and the validation cohort (41.3% *vs*. 8.3%; log-rank *P*<0.001) (**Figure 5**). Multivariate Cox regression analysis showed that the LM risk score was an independent predictor of LM, with an HR of 0.190 (95% CI: 0.121, 0.302; *P*<0.001) in the training cohort (**Table 2**). The time-dependent ROC curves indicated that the LM risk score had good discrimination in the training and validation cohorts (**Figure 6**).

**Table 2:** Univariate and multivariate Cox regression in the training cohort.

Variable	Univariate analysis		Multivariate analysis	
	HR (95% CI)	*P*	HR (95% CI)	*P*
**Age, years**	1.009 (0.992, 1.027)	0.294		
**Sex**				
Male	Ref			
Female	1.052 (0.691, 1.603)	0.812		
**Primary tumor location**				
Left-sided	Ref			
Right-sided	1.253 (0.791, 1.983)	0.336		
**Preoperative CEA level**				
Normal	Ref			
Elevated	1.423 (0.946, 2.142)	0.091	NA	NA
**Preoperative CA19-9 level**				
Normal	Ref			
Elevated	1.103 (0.666, 1.828)	0.703		
**VELIPI**				
No	Ref		Ref	
Yes	2.040 (1.339, 3.109)	0.001	1.589 (1.036, 2.438)	0.034
**Tumor differentiation**				
Well or moderately	Ref			
Poorly or undifferentiated	1.241 (0.756, 2.037)	0.393		
**KRAS**				
Wild type	Ref			
Mutation	1.196 (0.781, 1.832)	0.410		
**BRAF**				
Wild type	Ref			
Mutation	1.466 (0.641, 3.355)	0.365		
**BRAS**				
Wild type	Ref			
Mutation	1.759 (0.884, 3.501)	0.107		
**PIK3CA**				
Wild type	Ref			
Mutation	1.622 (0.958, 2.747)	0.072	NA	NA
**pT stage**				
I-II	Ref		Ref	
III	2.329 (1.382, 3.925)	0.001	1.747 (1.023, 2.983)	0.041
IV	2.873 (1.587, 5.201)	<0.001	2.022 (1.089, 3.755)	0.026
**pN stage**				
0	Ref		Ref	
I	2.417 (1.460, 4.000)	0.001	1.995 (1.196, 3.329)	0.008
II	4.943 (3.024, 8.080)	<0.001	2.885 (1.720, 4.841)	<0.001
**LM risk score**				
High	Ref		Ref	
Low	0.159 (0.102, 0.249)	<0.001	0.190 (0.121, 0.302)	<0.001

HR, hazard ratio; CI, confidence interval; Ref, reference; CEA, carcinoembryonic antigen; CA19-9, carbohydrate antigen 19-9; VELIPI, vascular emboli or lymphatic invasion or perineurial invasion; NA, not available.

Furthermore, patients with a low LM risk score had a significantly better 3-year DFS (83.6% *vs*. 51.9%; log-rank *P* < 0.001) and OS (91.2% *vs*. 63.2%; log-rank *P* < 0.001) than patients with a high LM risk score (**Supplementary Figures S5A, B**), and the HRs were 0.254 (95% CI: 0.174, 0.030) for DFS and 0.263 (95% CI: 0.167, 0.415) for OS. Similarly, this result was also presented in the validation cohort (**Supplementary Figures S5C, D**), and the corresponding HRs were 0.272 (95% CI: 0.159, 0.466) and 0.228 (95% CI: 0.114, 0.457) for DFS and OS, respectively.

### Development and Validation of the Nomogram

**Figure 7:**
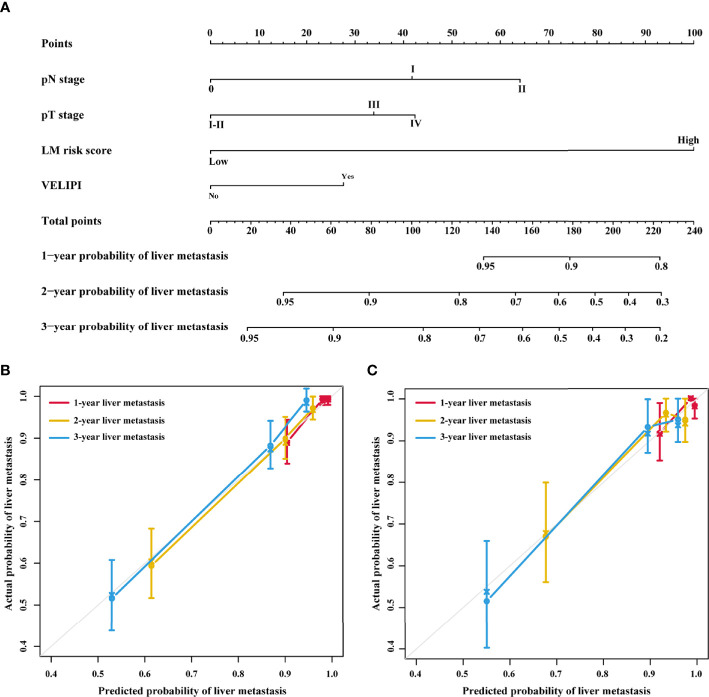
Integrated nomogram and the corresponding calibration curves. **(A)** Nomogram integrating the LM risk score, pT stage, pN stage and VELIPI for predicting LM. **(B)** Calibration curve of the integrated nomogram in the training cohort. **(C)** Calibration curve of the integrated nomogram in the validation cohort. VELIPI, vascular emboli or lymphatic invasion or perineurial invasion; LM, liver metastasis.

**Figure 8:**
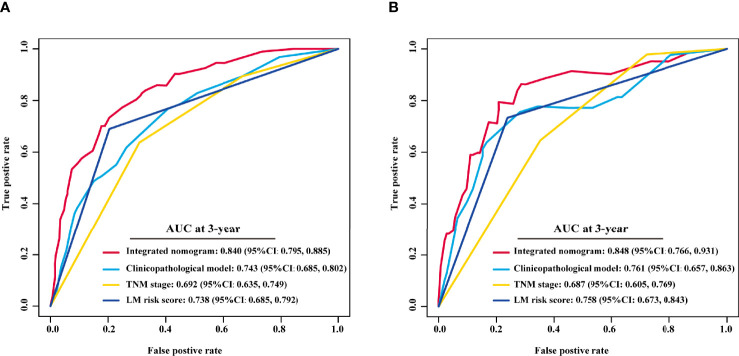
Comparison of the integrated nomogram and other models in the training and validation cohorts. The 3-year time-dependent ROC curves of the integrated nomogram, clinicopathological model, TNM stage and LM risk score alone in the training **(A)** and validation cohorts **(B)**. TNM, tumor-node-metastasis; AUC, area under the ROC curve; ROC, receiver operating characteristic; LM, liver metastasis; CI, confidence interval.

Univariate Cox analysis revealed that the LM risk score, preoperative CEA level, VELIPI, PIK3CA, pT stage, and pN stage were potential predictors of LM (*P*<0.010). The LM risk score, VELIPI, pT stage, and pN stage were identified as independent risk factors for LM according to the multivariate Cox analysis. Then, an integrated nomogram was developed based on the four variables (**Figure 7A**). The calibration curve showed good agreement between the predicted and actual probabilities of LM in the training cohort (**Figure 7B**) and the validation cohort (**Figure 7C**). The integrated nomogram achieved satisfactory discrimination, with a C-index of 0.807 (95% CI: 0.787, 0.827) and an area under the curve (AUC) of 0.840 (95% CI: 0.795, 0.885) at 3 years (**Figure 8A**) for predicting LM in the training cohort. In the validation cohort, the C-index was 0.812 (95% CI: 0.773, 0.850) and the AUC was 0.848 (95% CI: 0.766, 0.931) at 3 years (**Figure 8B**).

### Comparison With the Traditional Model

**Figure 9:**
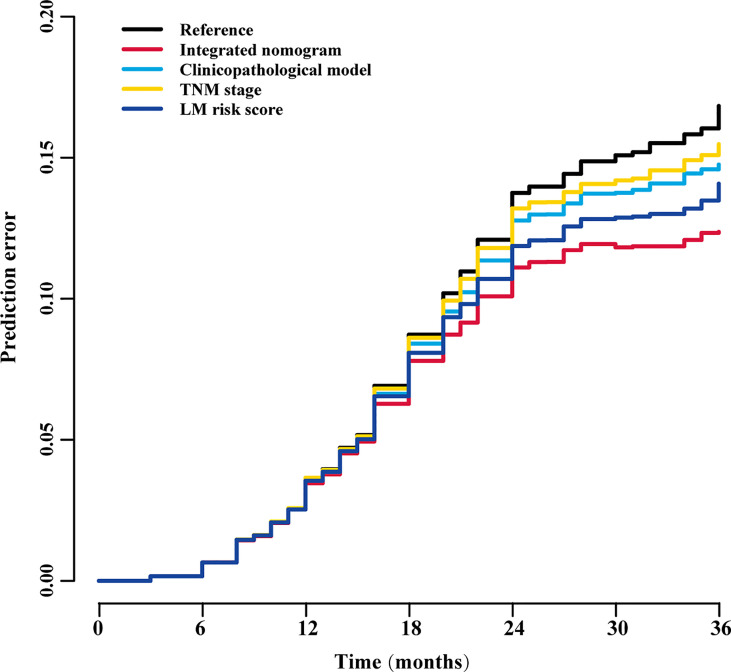
Prediction error curves for each model for stratifying liver metastasis in all patients. TNM, tumor-node-metastasis; LM, liver metastasis.

**Figure 10:**
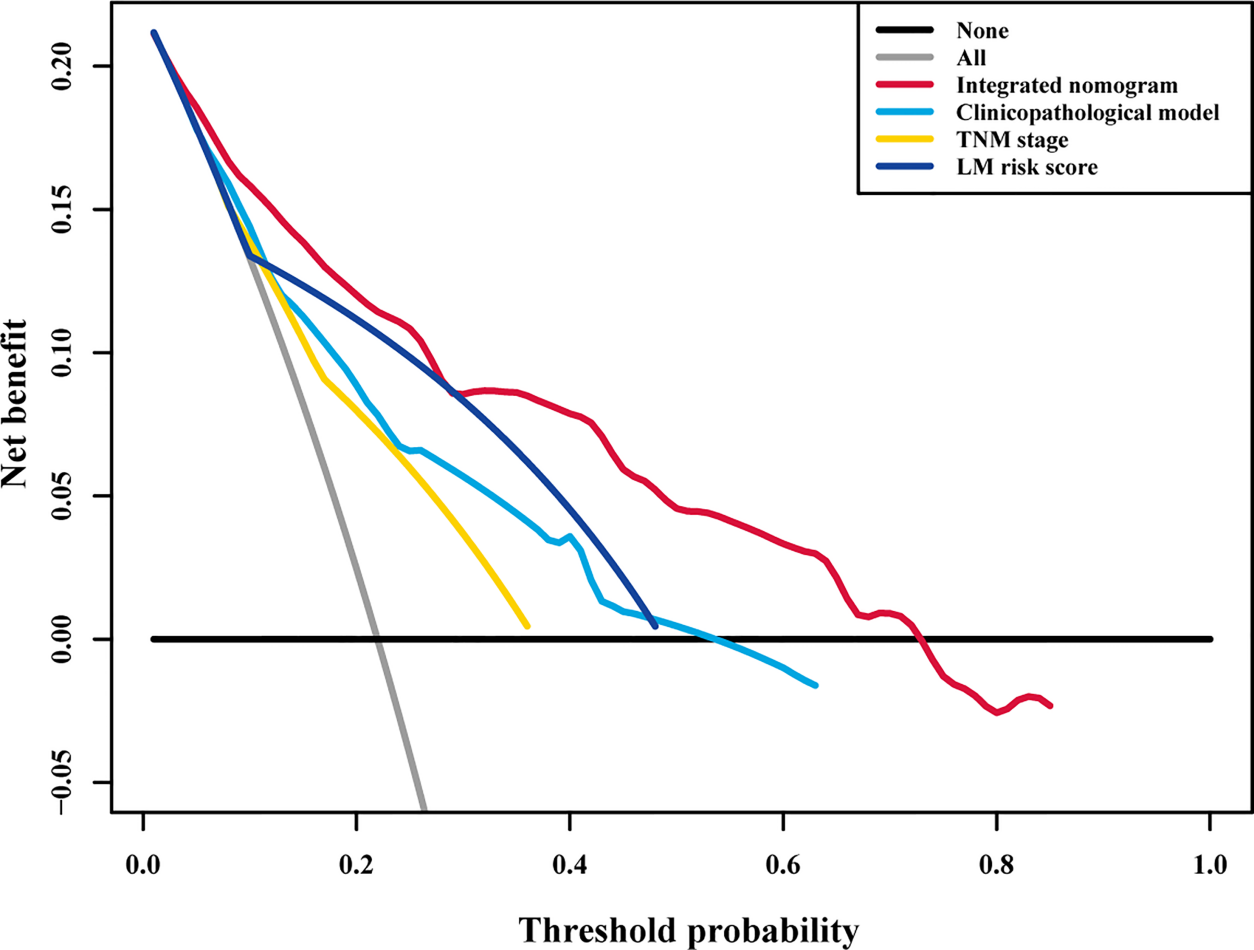
Decision curve analysis for each model in all patients. Decision curve analysis for predicting liver metastasis in all patients. The y-axis measures the net benefit, the red line represents the integrated nomogram, the blue line represents the clinicopathological model, the green line represents the LM risk score alone, the yellow line represents TNM stage, the black line represents the assumption that no patients developed liver metastasis, and the gray line represents the assumption that all patients developed liver metastasis. The net benefit was calculated by summing the benefits (true positive results) and subtracting the harms (false positive results), weighting the latter by a factor related to the relative harm of an undetected liver metastasis compared with the harm of unnecessary treatment. TNM, tumor-node-metastasis; LM, liver metastasis.

To assess the advantage of the integrated nomogram over the traditional model, we excluded the LM risk score and constructed a clinicopathological model based on VELIPI, pT stage, and pN stage (**Supplementary Table S2**). The clinicopathological model generated C-indexes of 0.716 (95% CI: 0.690, 0.743) and 0.741 (95% CI: 0.697, 0.785) in the training and validation cohorts, respectively. The integrated nomogram exhibited a higher C-index to predict LM than the clinicopathological model, TNM stage, and LM risk score alone in the two cohorts (all *P*<0.05) (**Table 3**). Moreover, the integrated nomogram also had a higher AUC at 3 years than the other models (**Table 4** and **Figure 8**). Furthermore, the integrated nomogram comprised of the clinicopathological model demonstrated an NRI of 0.480 (95% CI: 0.377, 0.582; *P*<0.001) and an IDI of 0.141 (95% CI: 0.075, 0.230; *P*<0.001) in the training cohort and an NRI of 0.504 (95%CI: 0.274, 0.648; *P* = 0.010) and an IDI of 0.135 (95%CI: 0.035, 0.249; *P*<0.001) in the validation cohort (**Table 5**), showing improved classification accuracy for predicting LM (**Supplementary Figure S6** and **Table 5**). The corresponding prediction error curves of all Cox models showed that the integrated nomogram obtained the lowest error compared to the other models (**Figure 9**). DCA revealed that if the threshold probability in the clinical decision was less than 88%, using the integrated nomogram to predict LM would add more net benefit than the other models (**Figure 10**), which indicated that the integrated nomogram has clinical application value.

**Table 3:** C-index comparison of the integrated nomogram with other prediction models.

Models	C-index (95% CI)	*P*
***Training cohort***		
Integrated nomogram	0.807 (0.787, 0.827)	Ref
Clinicopathological model	0.716 (0.690, 0.743)	<0.001
TNM stage	0.666 (0.641, 0.691)	<0.001
LM risk score	0.719 (0.696, 0.743)	<0.001
***Validation cohort***		
Integrated nomogram	0.812 (0.773, 0.850)	Ref
Clinicopathological model	0.741 (0.697, 0.785)	0.001
TNM stage	0.670 (0.635, 0.704)	<0.001
LM risk score	0.722 (0.686, 0.759)	<0.001

Ref, reference; CI, confidence interval.

**Table 4:** ROC comparison of the integrated nomogram with other prediction models at 3 years.

Models	AUC (95% CI)	*P*
***Training cohort***		
Integrated nomogram	0.840 (0.795, 0.885)	Ref
Clinicopathological model	0.743 (0.685, 0.802)	<0.001
TNM stage	0.692 (0.635, 0.749)	<0.001
LM risk score	0.738 (0.685, 0.792)	<0.001
***Validation cohort***		
Integrated nomogram	0.848 (0.766, 0.931)	Ref
Clinicopathological model	0.761 (0.657, 0.863)	0.004
TNM stage	0.687 (0.605, 0.769)	0.001
LM risk score	0.758 (0.673, 0.843)	0.002

ROC, receiver operating characteristic; CI, confidence interval; Ref, reference.

**Table 5:** Net reclassification and integrated discrimination improvement by comparing the integrated nomogram with the clinicopathological model.

Models	NRI (95% CI)	*P*	IDI (95% CI)	*P*
***Integrated nomogram vs. Clinicopathological model***				
Training cohort	0.480 (0.377, 0.582)	<0.001	0.141 (0.075, 0.230)	<0.001
Validation cohort	0.504 (0.274, 0.648)	0.010	0.135 (0.035, 0.249)	<0.001

NRI, net reclassification improvement; IDI, integrated discrimination improvement; CI, confidence interval.

## Discussion

The accurate prediction of metachronous LM is necessary for the selection of treatment strategies and the improvement of prognosis of stage I–III CRC patients after radical surgery. In this study, we constructed an LM risk score based on digital HE-stained images, and the ResNet-50 model was significantly associated with LM. The nomogram integrating the LM risk score, pT stage, pN stage, and VELIPI can precisely predict LM with satisfactory discrimination, calibration, and clinical application value.

Metachronous LM significantly impacts the prognosis of CRC patients who undergo radical surgery (39). The liver is the most common metastatic site of CRC, and 80% of LMs occur within two years after curative colectomy (5, 6, 40, 41). LM is the main cause of death in these patients. Hence, early detection and treatment can effectively improve the prognosis of metachronous LM patients. Accurately predicting which patients are at high risk and choosing treatment options are important clinical problems. Although the TNM staging system is widely used in clinical practice, it cannot sufficiently predict the risk of metachronous LM, and an effective biomarker is needed to supplement the TNM staging system.

With the development of full-slide digital scanning technology, all image information on traditional slides can be digitized to form a digitized slice, namely, a digital pathological image. It digitizes and networks pathological resources, realizing the permanent storage of visualized data. More importantly, digital HE images contain much potential pathological and prognostic information (32, 42). Recently, deep learning approaches have shown promise in tumor histopathological assessment (18, 19, 27). Compared with traditional image analysis methods, deep learning does not require professional knowledge to define several hand-made features. Deep learning can directly extract features related to the outcomes from the image, and this process is performed automatically. Hence, deep learning technology has been successfully applied to the analysis of digital HE images, such as the classification and localization of colon tissue (33) and the diagnosis of lung cancer (18). In addition, imaging genomics research, such as predicting microsatellite instability (MSI) status (34) and immune subtypes (13) from HE digital images of gastrointestinal cancer, suggests that digital HE images combined with deep learning is feasible to explore the characterization of the tumor microenvironment. Among several types of CNNs that have been proposed, ResNet has been widely used for deeper learning because it can effectively avoid gradient explosions. Hence, this study used ResNet-50 to analyze the relationship between HE images and LM in stage I-III CRC patients. We found that the LM risk score is an independent risk factor for LM, and patients with a high LM risk score were more likely to have postoperative LM than patients with a low LM risk score. An activation map was obtained, which can determine the tumor regions that the ResNet-50 model assigns high values in patients with a high risk of LM (**Supplementary Figure S3**). According to the activation map, in addition to the heterogeneity of tumor cells, the difference in the extracellular matrix and the tumor-stroma ratio may be related to the various probabilities of LM in stage I-III CRC patients (35, 36).

According to the results of multivariable Cox regression, the prediction model was constructed by integrating the LM risk score and clinicopathological predictors and then presented as an easy-to-use nomogram. The nomogram can visualize complex and abstract regression models and promote communication between doctors and patients (37, 38, 43). It is helpful for doctors and patients to jointly formulate individualized treatment strategies. T stage, N stage, and vascular invasion are recognized risk factors for metachronous LM (39, 44, 45), which is consistent with our results. To evaluate the incremental value of the LM risk score, we constructed a clinicopathological model. Then, we compared the integrated nomogram with the clinicopathological model, TNM stage, and LM risk score alone. The results showed that the integrated nomogram has better discrimination and calibration than other models, and DCA confirmed that the integrated nomogram has a higher clinical application value. Additionally, NRI and IDI showed that the integrated nomogram has the best accuracy. Therefore, the nomogram based on the LM risk score is significantly superior to traditional clinicopathological models. Based on the nomogram, we recommend that patients with a high risk of LM should undergo more rigorous postoperative monitoring and that adjuvant chemotherapy is essential.

Our research has the following advantages. First, HE staining of tumor resection specimens and then TNM staging are necessary processes for each patient, so they will not increase the financial burden of the patient or the workload of the pathologist; furthermore, all patients had undergone close follow-up for at least 3 years.

Although our work is stimulating, there are still some limitations. First, this study is a retrospective study, and selection bias cannot be avoided. Therefore, further prospective multicenter studies are needed to prove the robustness of the integrated nomogram. Second, the CNN-based model has a black-box nature, and we cannot use specific parameters to display the correlation between digital HE images and LM. Third, ROIs still needed to be manually selected, and we need to further optimize the deep learning model to realize the automatic annotation of ROIs. Fourth, the construction of the nomogram is a multistep process, the clinicopathological variables entering directly into the ResNet 50 model can enhance the efficiency and possibly even improve the performance of the model.

In conclusion, we found that the LM risk score based on ResNet-50 and digital HE images was significantly associated with LM. Furthermore, an integrated nomogram could identify stage I-III CRC patients at a high risk of developing LM after primary colectomy, which could serve as a potential tool to choose appropriate treatment to improve the survival of stage I-III CRC patients.

## Data Availability Statement

The raw data supporting the conclusions of this article will be made available by the authors, without undue reservation.

## Ethics Statement

The studies involving human participants were reviewed and approved by Institutional Review Boards of Hunan Provincial People’s Hospital and Chenzhou No. 1 People’s Hospital. The patients/participants provided their written informed consent to participate in this study.

## Author Contributions

CX, MZ, XY, HW, WJ, and JL conceived and designed the study. CX, MZ, XY, HW, ZT, ZZ, ZT, QL, XL, WJ, and JL acquired the data. WJ and JL verified the data. WJ and JL performed the quality control of the data. CX, MZ, XY, HW, WJ, and JL performed the statistical analyses. CX, MZ, XY, HW, WJ, and JL developed and validated the prediction model. CX, MZ, XY, HW, WJ, and JL prepared the first draft of the manuscript. All authors contributed to the article and approved the submitted version.

## Funding

This work was supported by grants from the Natural Science Foundation of Hunan Province (2018JJ2229) and Science & Technology Project of Chenzhou (zdyf201923).

## Conflict of Interest

The authors declare that the research was conducted in the absence of any commercial or financial relationships that could be construed as a potential conflict of interest.

## Publisher’s Note

All claims expressed in this article are solely those of the authors and do not necessarily represent those of their affiliated organizations, or those of the publisher, the editors and the reviewers. Any product that may be evaluated in this article, or claim that may be made by its manufacturer, is not guaranteed or endorsed by the publisher.

## Supplementary Material

Supplementary Figure 1 Kaplan–Meier survival analysis in the training and validation cohorts. DFS **(A)** and OS **(B)** curves in the training cohort. DFS **(C)** and OS **(D)** curves in the validation cohort. OS, overall survival; DFS, disease-free survival.

Supplementary Figure 2 Architecture and loss function of ResNet-50. The architecture of ResNet-50 is shown and includes convolution layers, max pooling layers, and a fully connected layer. ResNet-50, 50-layer residual network; LM, liver metastasis.

Supplementary Figure 3 Representative HE images of high and low LM risk scores. The activation maps for representative images of high and low risk scores, which reflect the weights corresponding to the liver metastasis risk, were obtained from the HE image. Class activation map for recurrence *vs*. non-recurrence. The high-intensity visual (red regions) area represents the area of interest that the model pays more attention to, which has important predictive value for liver metastasis. On the other hand, the blue area is the area that the model pays less attention to, which has little important predictive value for liver metastasis. LM, liver metastasis.

Supplementary Figure 4 Distribution of the LM risk score in the training and validation cohorts. The distribution of the LM risk score classified into the low- and high-LM risk score groups based on a cutoff value of 0.49 in the training cohort **(A)** and the validation cohort **(B)**. LM, liver metastasis.

Supplementary Figure 5 Relationship of the LM risk score with survival in the training cohort and validation cohort. Three-year DFS **(A)** and OS **(B)** comparison between the high- and low-LM risk score groups in the training cohort. Three-year DFS **(C)** and OS **(D)** comparison between the high- and low-LM risk score groups in the validation cohort. OS, overall survival; DFS, disease-free survival; HR, hazard ratio; LM, liver metastasis.

Supplementary Figure 6 Plots of net reclassification improvement in the training and validation cohorts. Net reclassification improvement by comparing the integrated nomogram with the clinicopathological model in the training **(A)** and validation cohorts **(B)**.

Supplementary Table 1 Clinical characteristics of the patients according to the LM risk score.

Supplementary Table 2 Univariate and multivariate Cox regression in the training cohort without the LM risk score.
