# Supplementary figures and images for: Accurate Prediction of Metachronous Liver Metastasis in Stage I-III Colorectal Cancer Patients Using Deep Learning With Digital Pathological Images
Source: Front Oncol. 2022 Apr 1;12:844067. doi: 10.3389/fonc.2022.844067 (PMC9010865; doi:10.3389/fonc.2022.844067)

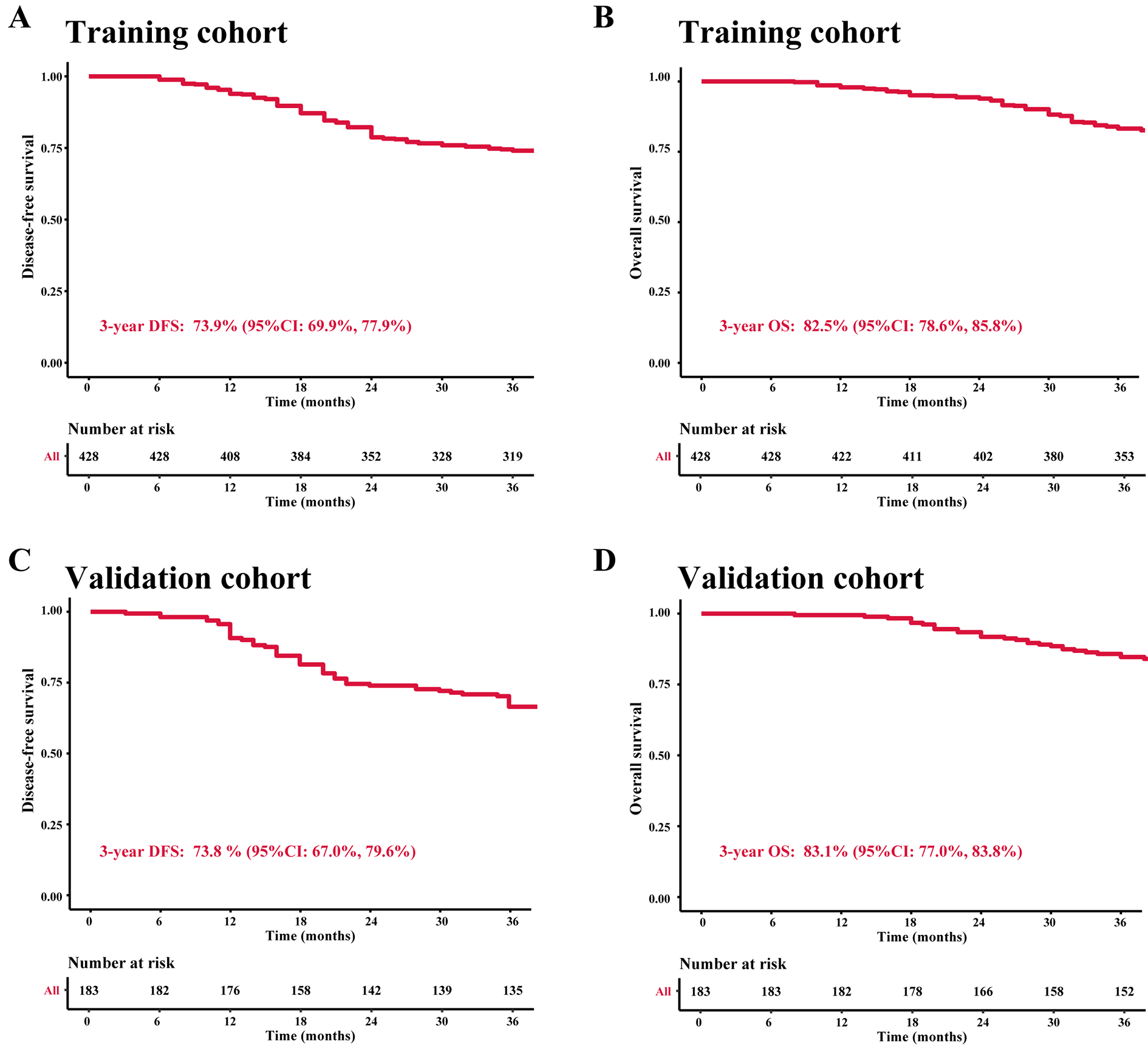

Supplement: Supplementary Figure 1 — Kaplan–Meier survival analysis in the training and validation cohorts. DFS (A) and OS (B) curves in the training cohort. DFS (C) and OS (D) curves in the validation cohort. OS, overall survival; DFS, disease-free survival. [file Image_1.tif]

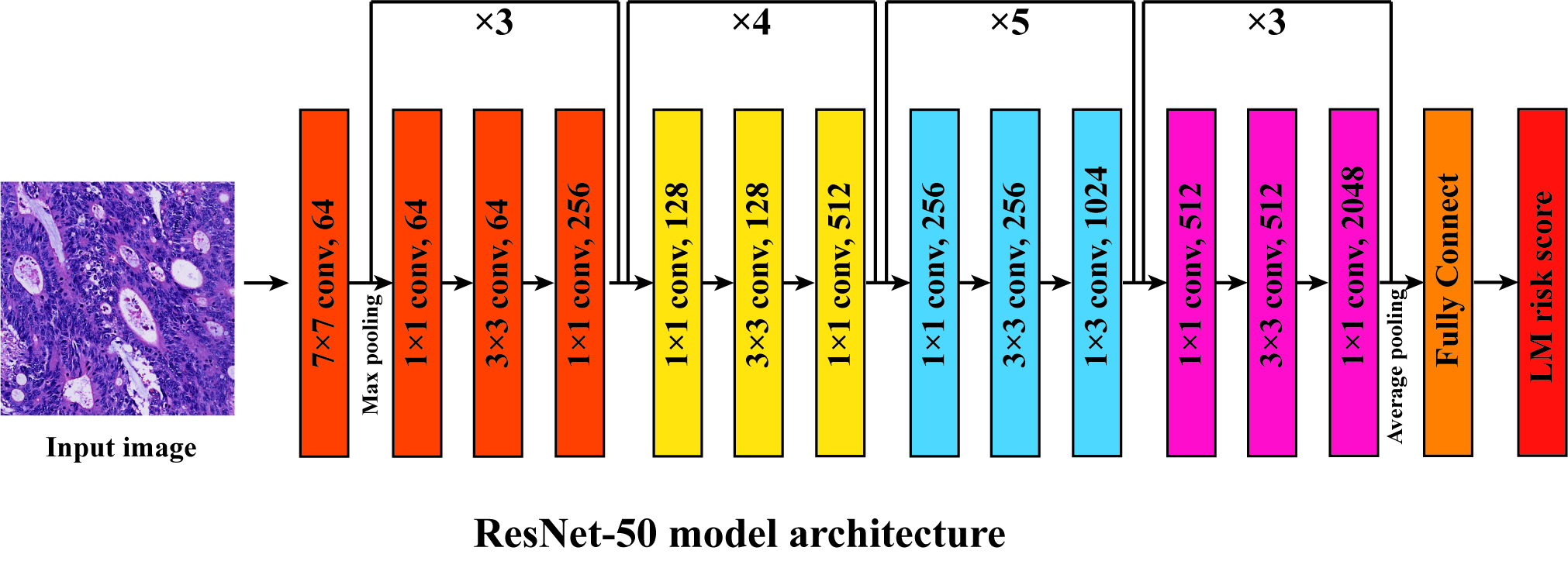

Supplement: Supplementary Figure 2 — Architecture and loss function of ResNet-50. The architecture of ResNet-50 is shown and includes convolution layers, max pooling layers, and a fully connected layer. ResNet-50, 50-layer residual network; LM, liver metastasis. [file Image_2.tif]

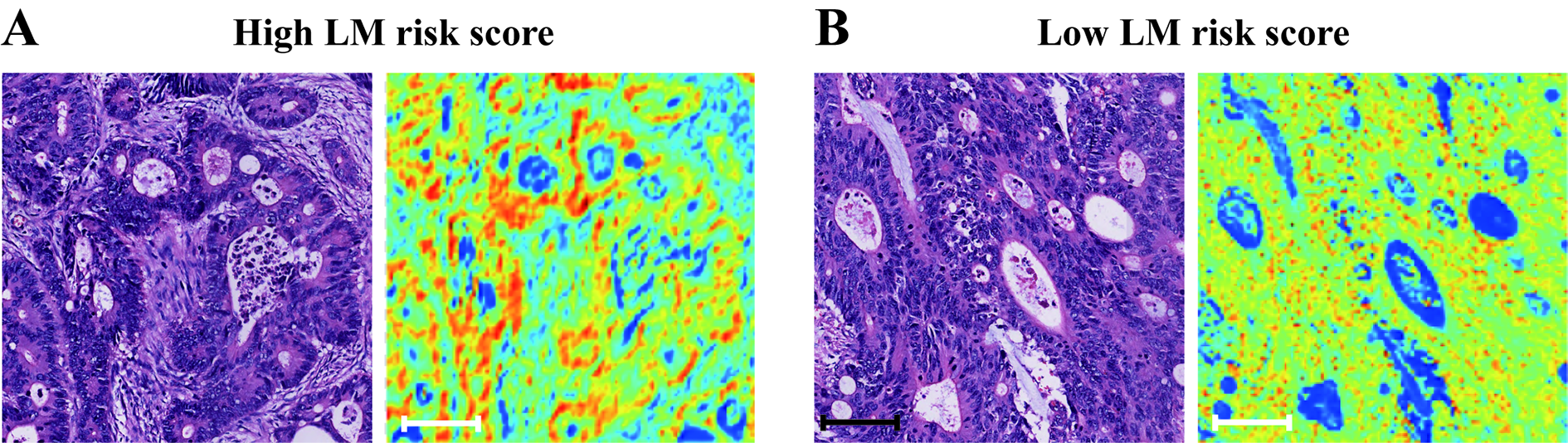

Supplement: Supplementary Figure 3 — Representative HE images of high and low LM risk scores. The activation maps for representative images of high and low risk scores, which reflect the weights corresponding to the liver metastasis risk, were obtained from the HE image. Class activation map for recurrence vs. non-recurrence. The high-intensity visual (red regions) area represents the area of interest that the model pays more attention to, which has important predictive value for liver metastasis. On the other hand, the blue area is the area that the model pays less attention to, which has little important predictive value for liver metastasis. LM, liver metastasis. [file Image_3.tif]

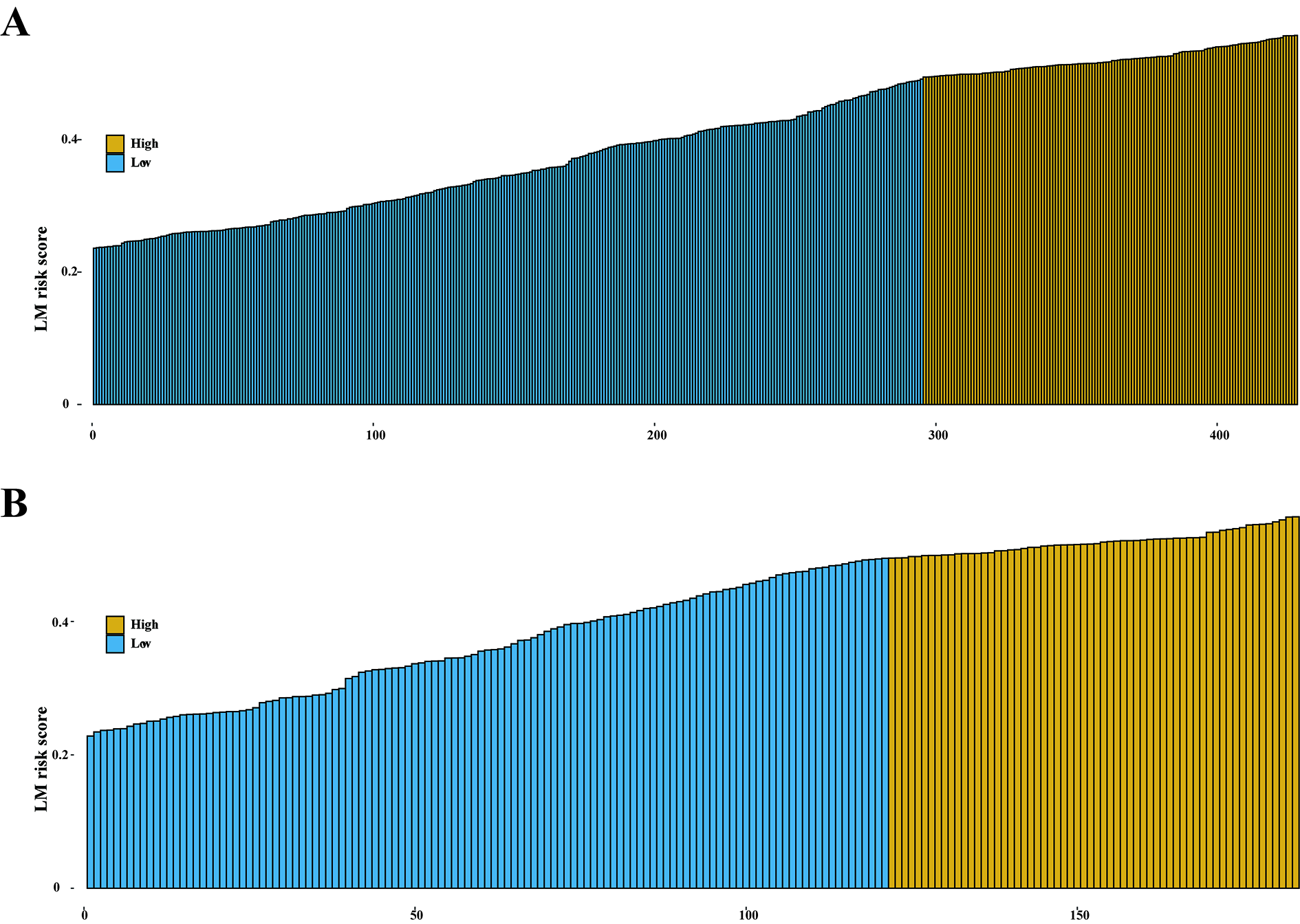

Supplement: Supplementary Figure 4 — Distribution of the LM risk score in the training and validation cohorts. The distribution of the LM risk score classified into the low- and high-LM risk score groups based on a cutoff value of 0.49 in the training cohort (A) and the validation cohort (B). LM, liver metastasis. [file Image_4.tif]

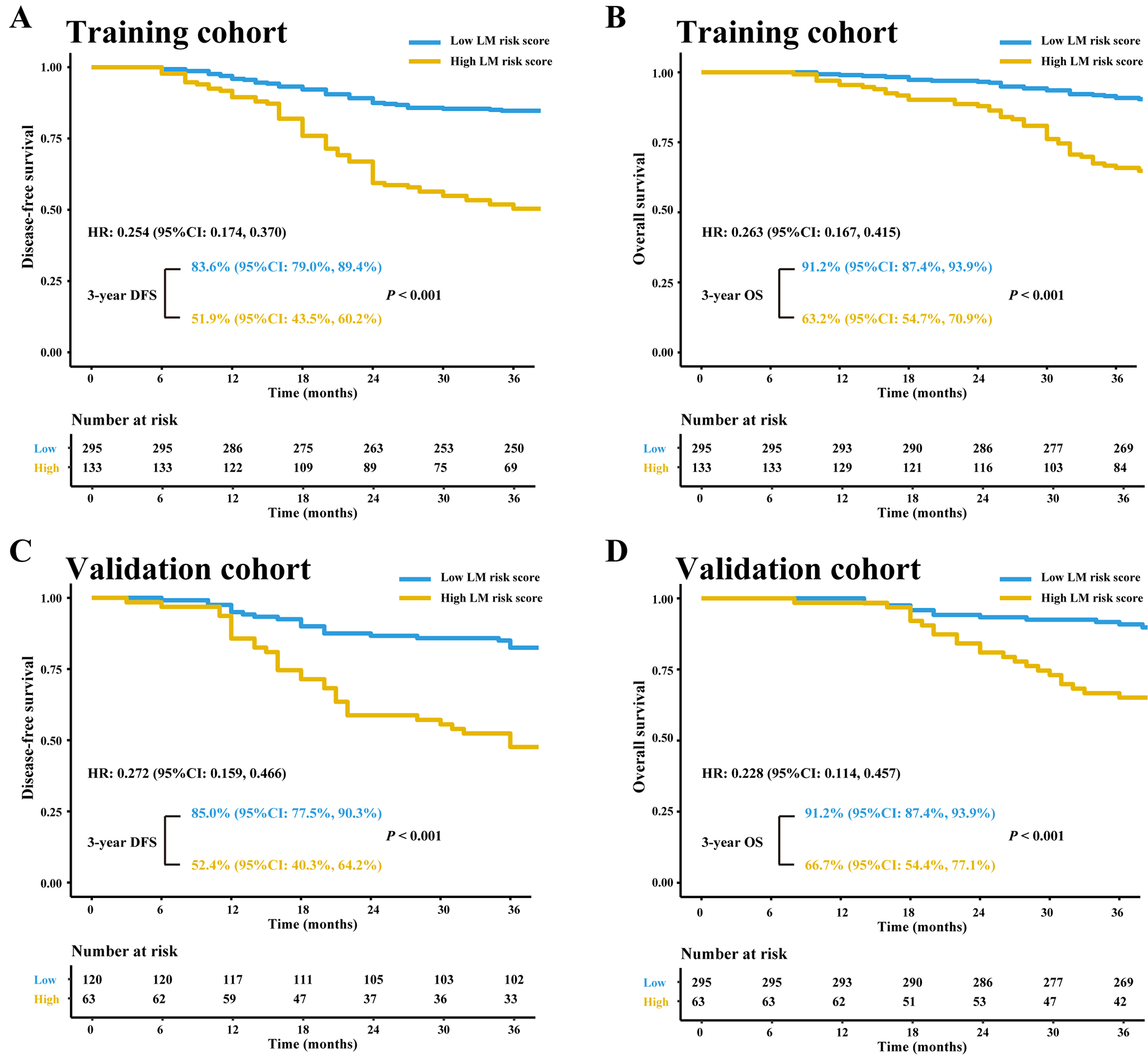

Supplement: Supplementary Figure 5 — Relationship of the LM risk score with survival in the training cohort and validation cohort. Three-year DFS (A) and OS (B) comparison between the high- and low-LM risk score groups in the training cohort. Three-year DFS (C) and OS (D) comparison between the high- and low-LM risk score groups in the validation cohort. OS, overall survival; DFS, disease-free survival; HR, hazard ratio; LM, liver metastasis. [file Image_5.tif]

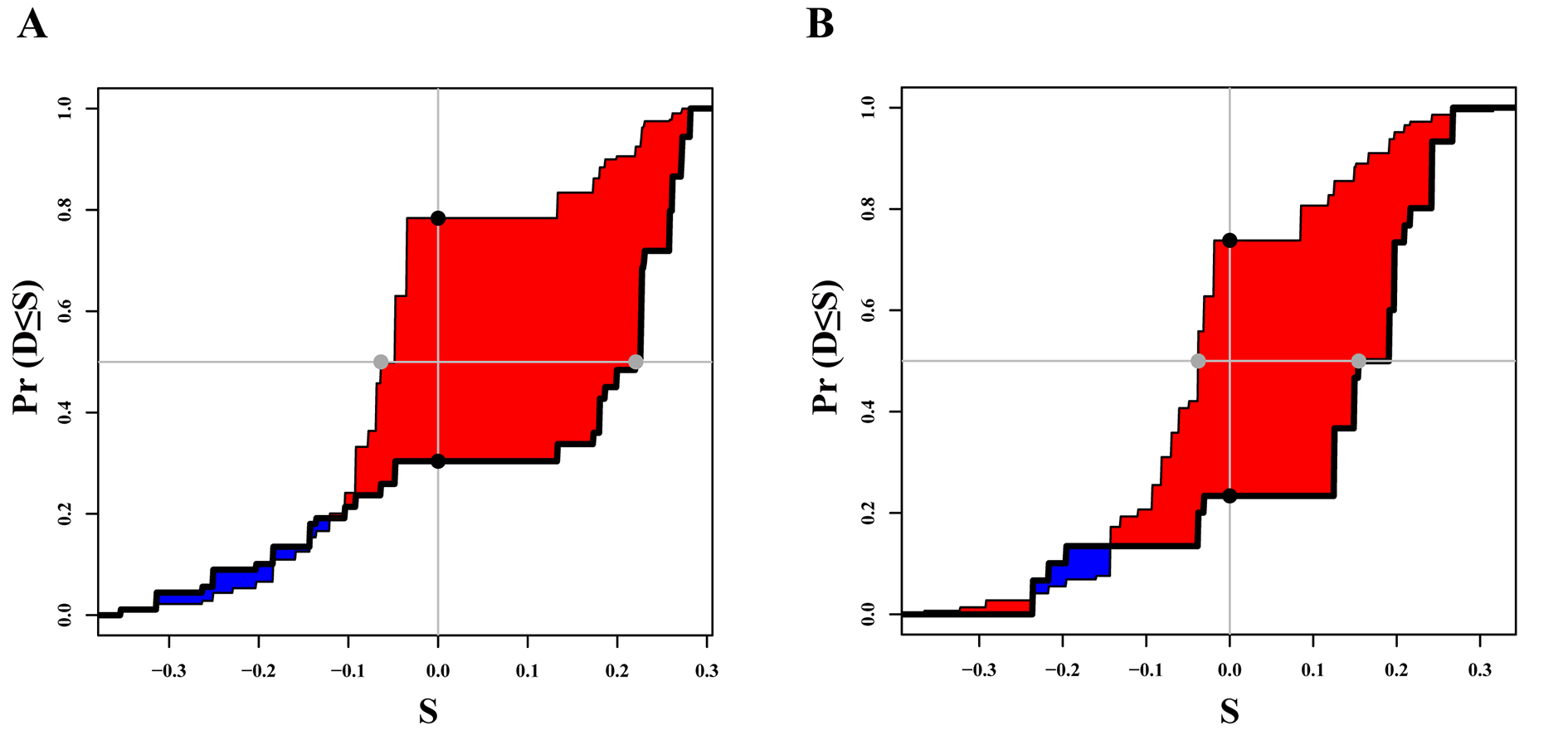

Supplement: Supplementary Figure 6 — Plots of net reclassification improvement in the training and validation cohorts. Net reclassification improvement by comparing the integrated nomogram with the clinicopathological model in the training (A) and validation cohorts (B). [file Image_6.tif]
