# Supplementary material for: Accurate Prediction of Metachronous Liver Metastasis in Stage I-III Colorectal Cancer Patients Using Deep Learning With Digital Pathological Images
Source: Front Oncol. 2022 Apr 1;12:844067. doi: 10.3389/fonc.2022.844067 (PMC9010865; doi:10.3389/fonc.2022.844067)
Supplement: Supplementary Table 1 — Clinical characteristics of the patients according to the LM risk score. [file Table_1.docx]

**TABLE S1. Clinical characteristics of the patients according to the LM risk score.**

| **Variable** | **Training cohort (N=428)** | | ***P*** | **Validation cohort (N=183)** | | ***P*** |
| --- | --- | --- | --- | --- | --- | --- |
|  | **Low**  **N=295 (%)** | **High**  **N=133 (%)** |  | **Low**  **N=120 (%)** | **High**  **N=63 (%)** |  |
| **Age, years** | 56.288 ± 11.848 | 57.594 ± 11.605 | 0.289 | 56.692 ± 12.832 | 54.873 ± 11.945 | 0.352 |
| **Sex** |  |  | 0.865 |  |  | 0.829 |
| Male | 186 (63.1) | 85 (63.9) |  | 80 (66.7) | 41 (65.1) |  |
| Female | 109 (36.9) | 48 (36.1) |  | 40 (33.3) | 22 (34.9) |  |
| **Primary tumor location** |  |  | 0.985 |  |  | 0.676 |
| Left-sided | 226 (76.6) | 102 (76.7) |  | 91 (75.8) | 46 (73.0) |  |
| Right-sided | 69 (23.40 | 31 (23.3) |  | 29 (24.2) | 17 (27.0) |  |
| **Preoperative CEA level** |  |  | 0.851 |  |  | 0.394 |
| Normal | 167 (45.6) | 74 (55.6) |  | 80 (66.7) | 38 (60.3) |  |
| Elevated | 128 (43.4) | 59 (44.4) |  | 40 (33.0) | 25 (39.7) |  |
| **Preoperative CA19-9 level** |  |  | 0.978 |  |  | 0.500 |
| Normal | 237 (80.3) | 107 (80.5) |  | 93 (77.5) | 46 (73.0) |  |
| Elevated | 58 (19.7) | 26 (19.5) |  | 27 (22.5) | 17 (27.0) |  |
| **VELIPI** |  |  | 0.152 |  |  | 0.005 |
| No | 164 (55.60 | 64 (48.1) |  | 79 (65.8) | 28 (44.4) |  |
| Yes | 131 (44.4) | 69 (51.9) |  | 41 (34.2) | 35 (55.6) |  |
| **Tumor differentiation** |  |  | 0.307 |  |  | 0.585 |
| Well or moderately | 243 (82.4) | 104 (78.2) |  | 95 (79.2) | 52 (82.5) |  |
| Poorly or undifferentiated | 52 (17.6) | 29 (21.8) |  | 25 (20.8) | 11 (17.5) |  |
| **KRAS** |  |  | 0.083 |  |  | 0.198 |
| Wild type | 209 (70.8) | 83 (62.4) |  | 89 (74.2) | 41 (65.1) |  |
| Mutation | 86 (29.2) | 50 (37.6) |  | 31 (25.8) | 22 (34.9) |  |
| **BRAF** |  |  | 0.135 |  |  | 1.000^a^ |
| Wild type | 264 (89.5) | 125 (94.0) |  | 113 (94.20 | 59 (93.7) |  |
| Mutation | 31 (10.5) | 8 (6.0) |  | 7 (5.8) | 4 (6.3) |  |
| **BRAS** |  |  | 0.583 |  |  | 0.071 |
| Wild type | 279 (94.6) | 124 (93.2) |  | 115 (95.8) | 56 (88.9) |  |
| Mutation | 16 (5.4) | 9 (6.8) |  | 5 (4.2) | 7 (11.1) |  |
| **PIK3CA** |  |  | 0.065 |  |  | 0.071 |
| Wild type | 263 (89.2) | 110 (82.7) |  | 107 (89.2) | 50 (79.4) |  |
| Mutation | 32 (10.8) | 23 (17.3) |  | 13 (10.8) | 13 (20.6) |  |
| **pT stage** |  |  | 0.280 |  |  | 0.425 |
| I-II | 121 (41.0) | 46 (34.6) |  | 48 (40.0) | 22 (34.9) |  |
| III | 126 (42.7) | 58 (43.6) |  | 56 (46.7) | 28 (44.4) |  |
| IV | 48 (16.3) | 29 (21.8) |  | 16 (13.30 | 13 (20.6) |  |
| **pN stage** |  |  | 0.003 |  |  | 0.303 |
| 0 | 195 (66.1) | 70 (52.6) |  | 74 (61.7) | 33 (52.4) |  |
| I | 67 (22.7) | 32 (24.1) |  | 29 (24.2) | 22 (34.9) |  |
| II | 33 (11.2) | 31 (23.3) |  | 17 (14.2) | 8 (12.7) |  |
| **TNM stage** |  |  | 0.029 |  |  | 0.409 |
| I | 89 (30.2) | 32 (24.1) |  | 27 (22.5) | 14 (22.2) |  |
| II | 106 (35.9) | 38 (28.6) |  | 47 (39.2) | 19 (30.2) |  |
| III | 100 (33.9) | 63 (47.4) |  | 46 (38.3) | 30 (47.6) |  |

Abbreviations: CEA, carcinoembryonic antigen; CA19-9, carbohydrate antigen 19-9; VELIPI, vascular emboli or lymphatic invasion or perineurial invasion.

a Fisher’s exact test
