# Supplementary material for: Accurate Prediction of Metachronous Liver Metastasis in Stage I-III Colorectal Cancer Patients Using Deep Learning With Digital Pathological Images
Source: Front Oncol. 2022 Apr 1;12:844067. doi: 10.3389/fonc.2022.844067 (PMC9010865; doi:10.3389/fonc.2022.844067)
Supplement: Supplementary Table 2 — Univariate and multivariate Cox regression in the training cohort without the LM risk score. [file Table_2.docx]

**TABLE S2. Univariate and multivariate Cox regression in the training cohort without the LM risk score.**

| **Variable** | **Univariate analysis** |  |  | **Multivariate analysis** |  |
| --- | --- | --- | --- | --- | --- |
|  | **HR (95% CI)** | ***P*** |  | **HR (95% CI)** | ***P*** |
| **Age, years** | 1.009 (0.992, 1.027) | 0.294 |  |  |  |
| **Sex** |  |  |  |  |  |
| Male | Ref |  |  |  |  |
| Female | 1.052 (0.691, 1.603) | 0.812 |  |  |  |
| **Primary tumor location** |  |  |  |  |  |
| Left-sided | Ref |  |  |  |  |
| Right-sided | 1.253 (0.791, 1.983) | 0.336 |  |  |  |
| **Preoperative CEA level** |  |  |  |  |  |
| Normal | Ref |  |  |  |  |
| Elevated | 1.423 (0.946, 2.142) | 0.091 |  | NA | NA |
| **Preoperative CA19-9 level** |  |  |  |  |  |
| Normal | Ref |  |  |  |  |
| Elevated | 1.103 (0.666, 1.828) | 0.703 |  |  |  |
| **VELIPI** |  |  |  |  |  |
| No | Ref |  |  | Ref |  |
| Yes | 2.040 (1.339, 3.109) | 0.001 |  | 1.790 (1.172, 2.735) | 0.007 |
| **Tumor differentiation** |  |  |  |  |  |
| Well or moderately | Ref |  |  |  |  |
| Poorly or undifferentiated | 1.241 (0.756, 2.037) | 0.393 |  |  |  |
| **KRAS** |  |  |  |  |  |
| Wild type | Ref |  |  |  |  |
| Mutation | 1.196 (0.781, 1.832) | 0.410 |  |  |  |
| **BRAF** |  |  |  |  |  |
| Wild type | Ref |  |  |  |  |
| Mutation | 1.466 (0.641, 3.355) | 0.365 |  |  |  |
| **BRAS** |  |  |  |  |  |
| Wild type | Ref |  |  |  |  |
| Mutation | 1.759 (0.884, 3.501) | 0.107 |  |  |  |
| **PIK3CA** |  |  |  |  |  |
| Wild type | Ref |  |  |  |  |
| Mutation | 1.622 (0.958, 2.747) | 0.072 |  | NA | NA |
| **pT stage** |  |  |  |  |  |
| I-II | Ref |  |  | Ref |  |
| III | 2.329 (1.382, 3.925) | 0.001 |  | 1.888 (1.114, 3.198) | 0.018 |
| IV | 2.873 (1.587, 5.201) | <0.001 |  | 2.004 (1.084, 3.704) | 0.027 |
| **pN stage** |  |  |  |  |  |
| 0 | Ref |  |  | Ref |  |
| I | 2.417 (1.460, 4.000) | 0.001 |  | 2.145 (1.290, 3.566) | 0.003 |
| II | 4.943 (3.024, 8.080) | <0.001 |  | 4.041 (2.434, 6.710) | <0.001 |

Abbreviations: HR, hazard ratio; CI, confidence interval; Ref, reference; CEA, carcinoembryonic antigen; CA19-9, carbohydrate antigen 19-9; VELIPI, vascular emboli or lymphatic invasion or perineurial invasion; NA, not available.
